# Antibody responses to *Chlamydia trachomatis* vaccine candidate antigens in *Chlamydia*-infected women and correlation with antibody-mediated phagocytosis of elementary bodies

**DOI:** 10.3389/fcimb.2024.1342621

**Published:** 2024-02-02

**Authors:** Hong Yu, William M. Geisler, Chuanbin Dai, Kanupriya Gupta, Gary Cutter, Robert C. Brunham

**Affiliations:** ^1^Department of Medicine, British Columbia Centre for Disease Control, University of British Columbia, Vancouver, BC, Canada; ^2^Department of Medicine, University of Alabama at Birmingham, Birmingham, AL, United States; ^3^Department of Biostatistics, University of Alabama at Birmingham, Birmingham, AL, United States

**Keywords:** *Chlamydia*, serology, antibody, antigen, Pgp3, IgG1 ELISA, antibody-mediated phagocytosis

## Abstract

Murine research has revealed a significant role for antibody responses in protection against *Chlamydia* reinfection. To explore potential humoral immune markers of protection elicited by *Chlamydia trachomatis* (CT) antigens in humans in the context of presumed clinical correlates of protection, we used both an IgG1-based ELISA and a conventional total IgG ELISA to evaluate antibody responses. We evaluated responses to five CT outer membrane proteins (PmpE, PmpF, PmpG, PmpH, and MOMP), along with other promising CT antigens (Pgp3 and HSP60), negative control antigens (RecO and AtpE), and CT elementary bodies (EBs) in sera from a well-characterized cohort of 60 women with different CT infection outcomes, including two outcomes that are likely clinical correlates of protective immunity: spontaneous resolution of infection and absence of reinfection after treatment. Furthermore, we used a flow cytometry-based assay to measure antibody-mediated phagocytosis by neutrophils in these sera. Results demonstrated that IgG1 ELISA displayed higher sensitivity than conventional total IgG ELISA in assessing antibody responses to CT EBs and antigens. Pgp3 IgG1 ELISA exhibited the highest sensitivity compared to IgG1 ELISA incorporating CT EBs or other antigens, confirming Pgp3 IgG1 ELISA as an ideal assay for CT antibody detection. Most (95%) sera from women with CT infection outcomes exhibited antibody-mediated phagocytosis of CT EBs, which was significantly correlated with IgG1 antibody responses to MOMP, Pgp3, HSP60, and PmpF. However, neither IgG1 responses to CT antigens and EBs nor antibody-mediated phagocytosis were associated with clinical correlates of protection. These findings suggest that neither CT IgG1 antibody detection nor antibody-mediated phagocytosis will be useful as immune correlates of protection against CT infection in humans.

## Introduction

*Chlamydia trachomatis* (CT) infection is a global health concern, being the most prevalent sexually transmitted bacterial infection, with over 128 million new cases estimated to occur annually (WHO, 2023), and having a major impact on women’s health because of CT-associated reproductive sequelae. While recommended antibiotics are effective against CT, most CT infections remain asymptomatic (Farley et al., 2003), and thus, are often undetected and untreated. Untreated CT infection can spread throughout the reproductive system, even when asymptomatic, resulting in significant complications in women, including pelvic inflammatory disease, ectopic pregnancy, and infertility (den Heijer et al., 2019). Additionally, CT increases the risk of HIV infection (Dzakah et al., 2022) and may act as a co-factor in the development of cervical (Bhuvanendran Pillai et al., 2022) and ovarian neoplasia (Paavonen et al., 2021).

Efforts of CT control programs have not diminished the high CT infection prevalence. In the U.S., CT infection rates have increased almost every year since CT infection became a nationally notifiable condition in 1995 (CDC, 2023). In British Columbia (BC), CT infection rates began to increase within a few years of a CT control program that was launched in 1991 (Brunham et al., 2005) and have continued to increase for most of the last decade, as has CT infection rates elsewhere in Canada (Zewude et al., 2022). An important change previously noted in CT infection epidemiology in BC was a 7-fold rise in CT reinfection rates in persons infected in the prior year (Brunham et al., 2005). This could be attributed to CT screening facilitating antibiotic treatment early in infection, which might hinder development of herd immunity against CT (as per the arrested immunity hypothesis) (Brunham and Rekart, 2008). Therefore, development of an effective CT vaccine is a global health priority in order to reduce CT infection rates and associated disease burden.

An important consideration in developing a CT vaccine for humans is determining the CT antigens to be included in the vaccine. Because of concerns that a CT EB vaccine could elicit immunopathology in humans and may not provide sufficient protective immunity, the focus of CT vaccine development has turned towards CT subunit vaccines (Yu et al., 2016; Zhong et al., 2019). Using an immunoproteomic approach, we identified five CT outer membrane proteins (OMPs), including four polymorphic membrane proteins (PmpE, PmpF, PmpG, PmpH) and the major outer membrane protein (MOMP) as CD4 T cell antigens (Karunakaran et al., 2008; Yu et al., 2012). These OMPs display immunogenic properties and provide partial protective immunity in mouse models (Yu et al., 2014; Karunakaran et al., 2015). Immunogenicity and protection induced by these CT OMPs in murine studies strongly support the need to extend immune studies of these CT OMPs to humans.

Another important component of CT vaccine development in humans is determining correlates of protection against CT in humans, both immune markers and clinical outcomes. Murine studies on *Chlamydia* natural history and immunity have demonstrated that only CD4+ T helper type 1 (Th1) responses (IFN-γ-mediated) are needed for clearance of initial infection and prevention of pathology, but both Th1 and antibody responses contribute to protection against reinfection (Morrison and Caldwell, 2002; Brunham and Rey-Ladino, 2005). Research revealed that antibodies play a crucial role in providing immunity against chlamydial genital tract reinfection. Moreover, the protection conferred by antibodies is highly dependent on CD4+ T cell-mediated adaptive changes that take place in the local genital tract tissues during the primary infection (Morrison and Morrison, 2005). Studies in humans have revealed that 11-44% of humans spontaneously cleared CT infection within a few weeks to a few months of diagnosis (before returning for treatment) (Geisler, 2010). We previously studied the impact of spontaneous clearance on CT reinfection risk in women and found it was associated with a 4-fold reduction in reinfection (Geisler et al., 2013). We have also shown that CT-specific CD4+ IFN-γ-responses are associated with lower reinfection rates in women (Bakshi et al., 2018).

Given that murine data indicate contribution of antibody responses to protection against *Chlamydia* reinfection, it is important to determine the role of antibody responses in protective immunity against CT in humans. To address this need, we used an IgG1-based ELISA (Geisler et al., 2012) and a conventional total IgG ELISA (Ness et al., 2003) to assess antibody responses to the five OMP potential CT vaccine antigens (MOMP, Pmp E, F, G and H) we previously studied in mice, along with other promising CT antigens (Pgp3 and HSP60) (Cohen et al., 2005; Galaleldeen et al., 2013), negative control antigens (RecO and AtpE), and CT EBs. We used sera from women who were part of a well-characterized cohort with different CT infection outcomes, including two outcomes that are probable clinical correlates of protective immunity (spontaneous resolution of infection and absence of reinfection), and from CT naïve women (i.e., negative controls). Furthermore, because neutrophils are a predominant cell population responding to genital *Chlamydia* infection (Kiviat et al., 1985) and murine studies that have shown that neutrophils but not NK cells are central to antibody-mediated protection against genital *Chlamydia* (Naglak et al., 2017), we employed a flow cytometry-based assay using PLB-815, neutrophils-like cells, to quantify antibody-mediated phagocytosis as a functional immune response to CT EBs. We assessed correlations between phagocytosis activity and IgG1 responses targeting both CT EBs and the CT antigens.

## Materials and methods

### Study population

The primary study population consisted of women (age ≥ 16) who were previously enrolled for a CT study as described elsewhere (Jordan et al., 2017; Bakshi et al., 2018). Briefly, they had a recent screening urogenital CT nucleic acid amplification test (NAAT) with a positive result and subsequently returned to an STD clinic in Birmingham, Alabama for treatment, at which time they were enrolled. At this enrollment visit (V1), they had CT NAAT repeated on an endocervical swab to determine the presence of either persisting infection (CT NAAT positive) or spontaneous resolution (CT NAAT negative), and both groups were treated under direct observation with azithromycin 1g single dose. Approximately 3 months later, they returned for a follow-up visit (V2), during which time a repeat endocervical CT NAAT was conducted to determine the presence or absence of reinfection. We also studied CT naïve women who were part of a previous study on the prevalence of CT infection in young women as described elsewhere (Gupta et al., 2021). Briefly, asymptomatic women ages 16-29 years who were seen in four clinical settings in Birmingham, Alabama, were enrolled for a single visit in which they were asked about prior CT infection and tested for active genital CT infection by NAAT and prior CT infection by evaluating for serum IgG1 antibody to CT EBs using ELISA.

As presented in **Table 1**, we classified women from the primary study population into three clinical outcome groups based on their CT NAAT results at V1 and V2: 1) “Spontaneous resolution” group: women who had spontaneously resolved CT infection before treatment (V1 CT NAAT negative) and showed no reinfection at follow-up (V2 CT NAAT negative), 2) “Persisting infection without reinfection” group: women who had a persisting infection at V1 (V1 CT NAAT positive) but exhibited no reinfection at V2 (V2 CT NAAT negative), and 3) “Persisting Infection with reinfection” group: women who had a persisting infection at V1 (V1 CT NAAT positive) and experienced reinfection at V2 (V2 CT NAAT positive). Women enrolled from the other cohort were determined to be CT naïve based on no prior reported history of CT infection, having a negative genital CT NAAT, and being CT seronegative. Antibody responses in sera from 20 women from each of the four groups (**Table 1**) were tested at Dr. Brunham’s laboratory at the University of British Columbia.

**Table 1:** Study groups and relation to *Chlamydia trachomatis* (CT) infection outcomes.

Study Group	Routine CT Screening	Baseline/Enrollment Visit (V1)	Follow-up Visit at 3 months (V2)
Spontaneous resolution	CT NAAT +	CT NAAT -	CT NAAT -
Persistent infection without reinfection	CT NAAT +	CT NAAT +	CT NAAT -
Persistent infection with reinfection	CT NAAT +	CT NAAT +	CT NAAT +
CT Naïve	N/A	CT NAAT - ; CT EB ELISA -	N/A

### Recombinant proteins (CT antigens)

Nine recombinant proteins from CT serovar D, PmpE (CT869_18–520aa_), PmpF (CT870_26-585aa_), PmpG (CT871_25-512aa_), PmpH (CT872_24–520aa_), MOMP (CT681_24-394aa_), HSP60 (CT110_1-544aa_), AtpE (CT310_1-208aa_), RecO (CT470_1-243aa_) and Pgp3 (pCT03_1-264aa_) were obtained from Biomatik (Cambridge, Ontario, Canada) as N-terminally His-Tagged proteins. PmpE, PmpF, PmpG, PmpH protein (N-terminal passenger domain without the signal peptide), MOMP protein (full-length protein without the signal peptide) and full-length proteins of HSP60, AtpE, RecO and Pgp3 were all produced in an *E. coli* expression system.

Each protein was received in lyophilized powder form with purity surpassing 95%, as verified through Coomassie blue-stained SDS-PAGE gel analysis, and endotoxin less than 0.1 EU/μg, determined by the Gel Clot Endotoxin Assay. The lyophilized proteins were finally resuspended in 1x phosphate-buffered saline (PBS) for use in the experiments.

### IgG1 ELISA

The nine CT recombinant proteins described above and CT EBs, a mixture of formalin-fixed CT serovars D, F and J, were used as antigens in the IgG1 ELISA. The preparation of the formalin-fixed EB mixture followed a previously established protocol (Geisler et al., 2012). 96-well polystyrene microtiter plates (Corning 3369) were coated with 100 μL of individual recombinant proteins (0.1 μg/well) or fixed CT EBs (2 x 10^5^ IFU/well) in 0.1 M carbonate-bicarbonate buffer (PH 9.6) overnight at 4°C and then were washed 3 times with PBS with 0.05% Tween 20 (PBST). Nonspecific binding sites were blocked by addition of 150 μL of 3% bovine serum albumin (BSA) in PBS to each well, for 2 h at 37°C. After 3 washings with PBST, 100 μL of human serum diluted to 1:32 in PBS with 0.5% BSA were added, in triplicate, and plates were incubated overnight at 4°C. A pool of Alkaline Phosphatase (AP)-conjugated mouse anti-Human IgG1 Fc fragment-specific monoclonal antibody (Clone HP6069, Millipore Sigma Cat# 401459) and AP-conjugated mouse anti-human IgG1 hinge monoclonal antibody (Clone 4E3, SouthernBiotech Cat# 9052-04) were used as secondary antibodies in this assay. After 3 washings with PBST, 100 μl of these secondary antibodies diluted to 1:500 in PBS with 0.5% BSA were added to each well and incubated for 2 hours at 37°C. After incubation, the plates were washed 4 times with PBST, and 100 μl of SIGMAFAST p-nitrophenyl phosphate substrate (p-NPP, Sigma N1891) was added to each well, followed by a 30-minute incubation at room temperature in the dark. The optical density (OD) of the enzymatic reaction was measured at 405 nm.

The cut-off value of a specific CT antigen for positive serologic responses was determined as mean + 2SD of OD value by antigen-specific IgG1 ELISA using serum samples from CT naïve women (CT Naive group in **Table 1**).

### Total IgG ELISA

Microplates were coated with the nine CT antigens and CT EBs and were blocked with 3% BSA in PBS. The procedures of microplate coating and blocking were akin to those in the IgG1 ELISA described above. Then, 100 μl of human serum diluted to 1:250 in PBS with 0.5% BSA was added, in triplicate, and plates were incubated overnight at 4°C. After 3 washings with PBST, 100 μl of horseradish peroxidase (HRP)–conjugated donkey anti-human IgG secondary antibody (Jackson ImmunoResearch, Cat# 709-035-149) diluted to 1:2000 in PBS with 0.5% BSA were added and incubated for 2 hours at 37°C in the dark. After 4 washings with PBST, 100 μl of 2,2-azino-bis (3-ethylbenzthiazoline-6-sulfonic acid) substrate was added. The plates were then incubated for 5 minutes at room temperature. The OD values of the enzymatic reaction were measured at 405 nm.

### Phagocytosis assay

The assay is a flow cytometry-based method to measure antibody-mediated phagocytosis and the methodology has been reported (Grasse et al., 2018). Briefly, CFSE-labeled CT EB serovar D was pre-incubated with heat-inactivated serum at 1:10 dilution for 40 min at 37°C and incubated for 3 h with DMF-treated (N,N-Dimethylformamide, Sigma Cat# 2650) PLB-985 cells (MOI 10) at 37°C, 5% CO_2_. PLB-985 cells (DSMZ no. ACC 139), an immature myeloid cell line, were differentiated by 100 mM DMF treatment for 5 days into terminally mature neutrophils. The uptake of antibody-coated bacteria by PLB-985 cells was measured on a flow cytometer (BD FACSVerse). CFSE-positive cells were gated as phagocytosing cells. The percent of phagocytosing cells at a 1:10 dilution of serum was recorded.

The cut-off value of phagocytosis percentage for positive serologic responses was determined as mean + 2SD of phagocytosis percentage value using the same 20 serum samples from CT naïve women as was used for the ELISA experiments described above.

### Statistical analysis

All data were analyzed using JMP PRO 16.0.0 (SAS Institute Inc., Cary, NC). The paired t-test was employed to compare IgG1 antibody OD values and phagocytosis activity percentages between two time points (V1 and V2). The unpaired t-test was used to compare IgG1 antibody OD values and phagocytosis activity percentages between any two study groups (CT outcome groups or CT Naïve group). Pearson’s correlation (tested with a t-test) was used to assess for correlation between phagocytosis and IgG1 responses for each of the 9 CT antigens or CT EBs. P values < 0.05 were considered significant.

## Results

### IgG1 ELISA to CT antigens and EBs demonstrated significantly higher OD values in sera from women with CT infection outcomes compared to the conventional total IgG ELISA method while maintaining a lower background signal in sera from CT-naïve women

Most existing CT serologic assays face a significant challenge due to their low to moderate sensitivity. Geisler et al. previously introduced an IgG1- and IgG3-based ELISA method designed for the sensitive and specific detection of anti-EB antibody responses (Geisler et al., 2012), with IgG1 reflecting a longer-lived antibody response.

**Figure 1:**
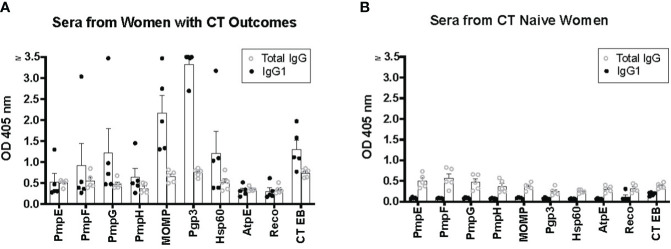
Enhanced sensitivity of IgG1 ELISA for detecting antibody responses to *Chlamydia trachomatis* (CT) antigens compared to total IgG ELISA. Five serum samples from five women with CT infection outcomes **(A)** and serum samples from five CT naïve women **(B)** were utilized for both IgG1 ELISA and total IgG ELISA.

We compared IgG1 ELISA with conventional total IgG ELISA for the nine CT antigens and CT EBs. We randomly selected five serum samples from women with CT infection outcomes and five serum samples from CT naïve women for a head-to-head comparison. The results revealed that for serum samples from women with CT infection outcomes, IgG1 ELISA produced higher OD values for immunoreactive CT antigens compared to total IgG ELISA (**Figure 1A**). Conversely, for serum samples from CT naïve women, IgG1 ELISA yielded lower OD values for all tested CT antigens than total IgG1 ELISA (**Figure 1B**). For instance, the OD value for antibody to Pgp3 in sera from women with CT infection outcomes was 3.33 ± 0.357 (mean ± SD) for IgG1 and 0.748 ± 0.0976 for total IgG, while in serum samples from CT naïve women, it was 0.0814 ± 0.0149 for IgG1 and 0.246 ± 0.0931 for total IgG. These results confirm the superior sensitivity and likely higher specificity of the IgG1 ELISA, characterized by higher OD values observed in sera from women with CT infection outcomes and lower background noise in sera from CT naïve women compared to total IgG ELISA. Consequently, we selected IgG1 ELISA for detection of antibody responses to CT antigens in our further antibody studies.

### IgG1 seropositivity rates were high in response to CT EBs and most of the nine CT antigens in women with CT infection outcomes

We analyzed 120 serum samples obtained from the V1 and V2 visits of the 60 women with CT infection outcomes to measure IgG1 responses to these antigens. As illustrated in **Table 2**, the frequency of IgG1 seropositivity to the nine CT antigens and CT EBs in order of highest to lowest frequency were as follows: Pgp3 (97.8%), CT EBs (95.8%), MOMP (90.8%), HSP60 (87.5%), PmpG (63.3%), PmpF (61.7%), PmpH (61.7%), RecO (45.8%), PmpE (34.2%), and AptE (23.3%).

**Table 2:** The frequency of seropositivity to 9 CT antigens and CT EB in 120 serum samples from women with CT infection outcomes determined by IgG1 ELISA.

CT Antigen	PmpE	PmpF	PmpG	PmpH	MOMP	Pgp3	HSP60	RecO	AtpE	CT EB
**Minimum OD**	0.098	0.094	0.109	0.105	0.18	0.109	0.079	0.103	0.083	0.27
**Median OD**	0.253	0.316	0.415	0.315	1.19	3.45	0.607	0.216	0.192	1.32
**Maximum OD**	2.61	3.04	3.47	2.81	3.5	3.5	3.33	1.36	1.99	2.58
**Mean OD**	0.38	0.473	0.572	0.509	1.57	2.97	0.801	0.289	0.28	1.32
Cut-off value*	0.236	0.267	0.321	0.248	0.428	0.256	0.210	0.230	0.274	0.392
**Positive Count**	41	74	76	74	109	117	105	55	28	115
**Seropositivity %**	34.2%	61.7%	63.3%	61.7%	90.8%	97.5%	87.5%	45.8%	23.3%	95.8%

*The cut-off value for a specific CT antigen was determined as mean + 2SD of OD value by antigen-specific IgG1 ELISA using 20 serum samples from CT naive women.

ELISA assays measuring antibodies to Pgp3, MOMP, HSP60, and CT EBs all elicited an exceptionally high seropositivity rate in women with CT outcomes, reflecting robust immunogenic antibody responses to these antigens in those with natural CT infection. Moreover, the majority of sera also demonstrate seropositivity to PmpG, PmpF, and PmpH, suggesting that these outer membrane proteins also possess immunogenic properties in women with natural CT infection.

### Pgp3-based IgG1 ELISA displayed superior sensitivity in comparison to MOMP-, CT EB-, and HSP60 IgG1 ELISAs

**Figure 2:**
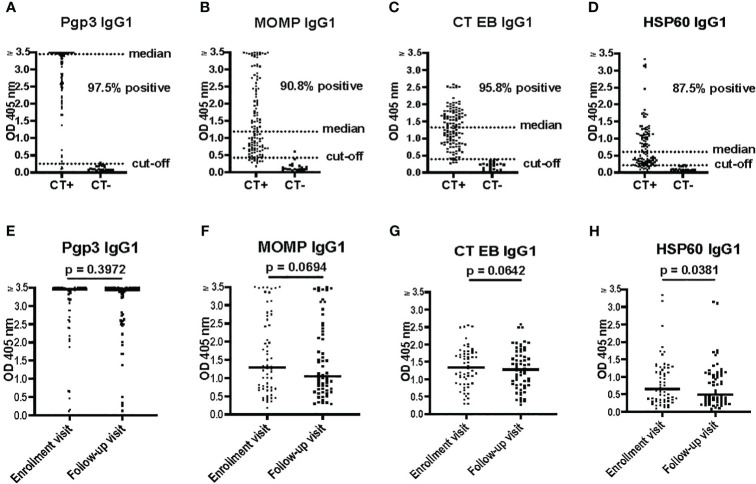
Pgp3 IgG1 ELISA is more sensitive for *Chlamydia trachomatis* (CT) antibody detection compared to HSP60 IgG1, MOMP IgG1, and CT EB IgG1 ELISAs. **(A–D)** Antibody responses to Pgp3, MOMP, CT EB or HSP60 determined by IgG1 ELISA in 120 serum samples (enrollment and follow-up) from 60 women with CT infection outcomes (CT+) and 20 serum samples from CT-naïve women (CT-). **(E–H)** Antibody responses to Pgp3, MOMP, CT EB or HSP60, at enrollment visit versus follow-up visit (paired t test).

Pgp3, MOMP, HSP60, and CT EBs are among the most commonly used antigens in various assays to detect CT antibodies and they have been reported as T cell antigen markers of protective immunity in humans (Cohen et al., 2005; Barral et al., 2014; Bakshi et al., 2018). In this study, sera from women with CT infection outcomes that were tested with the Pgp3-based IgG1 ELISA not only demonstrated the highest seropositivity rates (97.5%) but also yielded significantly higher OD values (median of 3.45) and comparatively lower background levels (cut-off of 0.256) in comparison to MOMP-based IgG1 ELISA (median of 1.19, cut-off of 0.428), CT EB-based IgG1 ELISA (median of 1.32, cut-off of 0.392), and HSP60-based IgG1 ELISA (median of 0.607, cut-off of 0.210) (**Figures 2A–D**).

No significant differences were observed in the magnitude of the IgG1 response among the tested CT antigens at V1 versus V2, except for HSP60 (p = 0.0381) (**Figures 2E–H**). The magnitude of the HSP60 IgG1 response exhibited a significant decrease between V1 and V2 in both the persisting infection without reinfection group (p = 0.0289) and the spontaneous resolution group (p = 0.0104), but not in the persisting infection with reinfection group (p = 0.7816) (data not shown). Therefore, sustained levels of HSP60 IgG1 antibodies may be an indicator of impaired immune protection to CT, although the mechanism for this is unknown.

### Pgp3 IgG1 ELISA is the most sensitive of the assays evaluated for CT antibody detection, with a higher magnitude of Pgp3 IgG1 response in current CT infection

**Figure 3:**
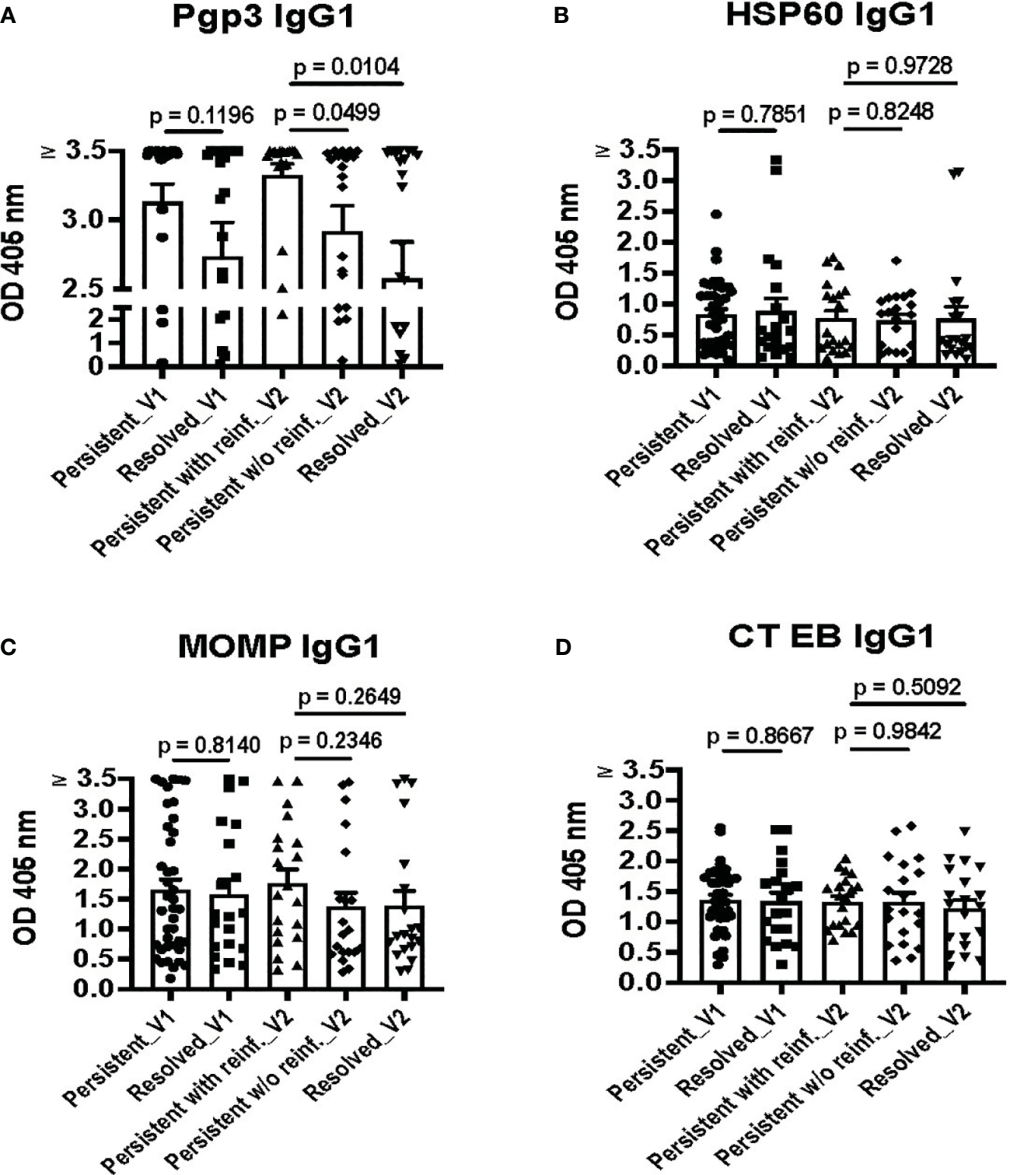
Pgp3 IgG1 ELISA is highly sensitive, with the strongest Pgp3 IgG1 responses during current *Chlamydia trachomatis* (CT) infection. IgG1 antibody responses to Pgp3 **(A)**, HSP60 **(B)**, MOMP **(C)**, and CT EBs **(D)** in the combined persisting infection groups at the enrollment visit (Persisting_V1), the spontaneous resolution group at the enrollment visit (Resolved_V1), the persisting infection with reinfection group at the follow-up visit (Persisting with reinf._V2), the persisting infection without reinfection group at the follow-up visit (Persisting w/o reinf._V2) and the spontaneous resolution group at the follow-up visit (Resolved_V2). (Unpaired t test between groups with different outcomes).

To identify possible immune correlates of protection conferred by antibodies against CT in humans in the context of presumed clinical correlates of protection, we analyzed IgG1 responses to the nine CT antigens and CT EBs in women with different CT infection outcomes. As shown in **Figure 3A**, the magnitude of the Pgp3 IgG1 response at V1 was somewhat higher in the combined persisting infection groups compared to the spontaneous resolution group (p = 0.1196). At V2, the magnitude of the Pgp3 IgG1 response in the persisting infection with reinfection group was significantly higher in comparison to both the persistent infection without reinfection group (p = 0.0499) and the spontaneous resolution group (p = 0.0104). However, there were no significant differences observed in the magnitude of HSP60 IgG1, MOMP IgG1, and CT EB IgG1 responses among these different groups (**Figures 3B–D**). These findings suggest that stronger Pgp3 IgG1 responses correlate with current CT infection status, which also implies that the magnitude of Pgp3 IgG1 responses partially decline with infection clearance and may be boosted with repeat infection.

Overall, we did not detect a higher magnitude of IgG1 responses to any of the 9 CT antigens or EBs in the spontaneous resolution group at V1 or the persisting infection without reinfection group (absence of reinfection) at V2, the two outcomes that are presumed clinical correlates of protective immunity. These results suggest that while detection of CT IgG1 antibodies may be useful for determining evidence of current or past CT infection, CT IgG1 antibody detection may not be useful as an immune correlate of protection to CT in humans.

### Antibody-mediated phagocytosis of CT EBs was detected in the vast majority of women with CT infection outcomes

**Figure 4:**
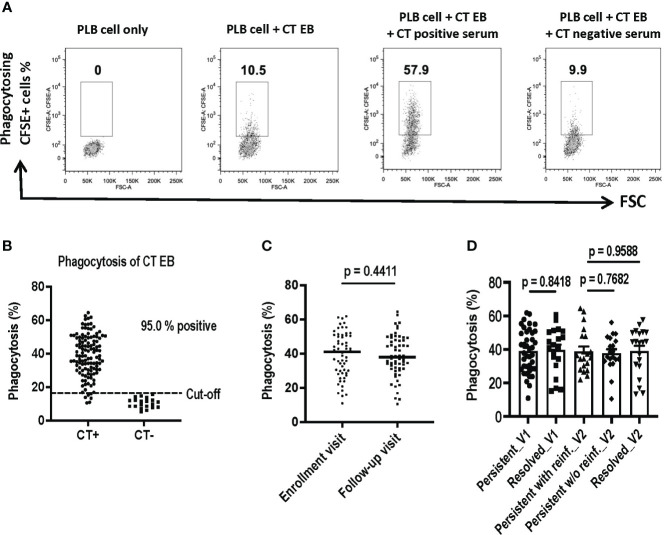
Assessment of Antibody-Mediated Phagocytosis of *Chlamydia trachomatis* (CT) EBs Using a Flow Cytometry-Based Assay. CFSE-labeled CT EBs were preincubated with human serum samples, followed by incubation with DMF-stimulated PLB-985 cells (neutrophil-like cells). The CFSE signal was quantified using flow cytometry. Cells were gated based on CFSE-positive (indicating phagocytosis) events. **(A)** Flow cytometry plots depict phagocytosing CFSE+ cells from PLB cells alone, PLB cells with CT EBs, and PLB cells with CT EBs plus serum samples from representative women with CT infection outcomes (CT+) or who are CT-naïve (CT-). **(B)** Percentage of phagocytosing CFSE+ cells in serum from 120 women with CT infection outcomes and 20 CT naïve women. **(C)** Percentage of phagocytosing CFSE+ cells at enrollment visit versus follow-up visit. (paired t test). **(D)** Percentage of phagocytosing CFSE+ cells in groups with different outcomes of CT infection (Unpaired t test).

We utilized a flow cytometry-based assay to quantify antibody-mediated phagocytosis of CT EBs as a functional antibody immune response. The assay is a simple and rapid method to measure capacity of antibodies to mediate Fc-receptor dependent phagocytosis of CT EBs. CFSE-labeled CT EBs were pre-incubated with sera samples, followed by co-incubation with PLB-985 cells (neutrophil-like cells). After uptake of antibody-coated bacteria, CFSE-positive PLB-985 cells were gated as phagocytosing cells. As depicted in **Figure 4A**, the flow cytometry plots illustrated the percentage of phagocytosing CFSE+ cells from PLB cells alone (0%), PLB cells with CT EBs (10.5%), and PLB cells with CT EBs plus sera from women with CT infection outcomes (57.9%) or CT naïve women (9.9%), thereby demonstrating a clear discrimination between women with CT infection outcomes vs. CT naïve women.

We assessed phagocytosis of CT EBs using 120 serum samples obtained from the V1 and V2 visits of the 60 women with CT infection outcomes. Serum samples were included from the 20 CT naïve women to determine the cut-off value (mean + 2SD). Our findings revealed that 95% of sera from women with CT infection outcomes exhibited positive antibody-mediated phagocytosis of CT EBs (**Figure 4B**). However, there was no significant difference in phagocytosis levels between V1 and V2 (**Figure 4C**) or among groups with different CT infection outcomes (**Figure 4D**). Thus, while serum antibody-mediated phagocytosis of CT EBs may serve as a sensitive marker for current or recent CT infection, it may not be useful as an immune correlate of protection to CT in humans.

### Antibody-mediated phagocytosis of CT EBs significantly correlated with IgG1 responses against selected CT antigens and EBs in women with CT infection outcomes

**Figure 5:**
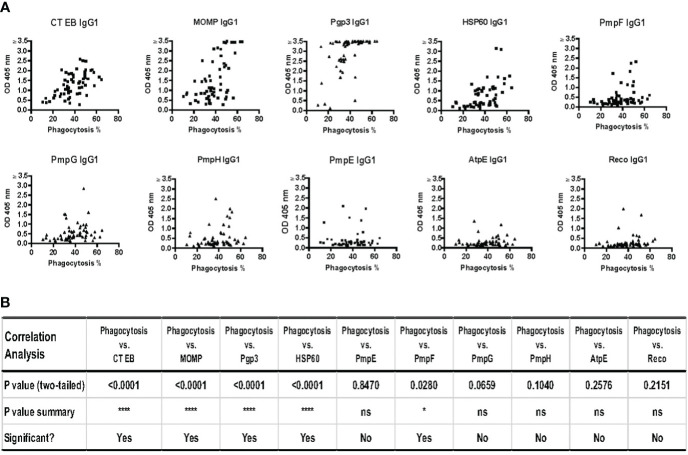
Strong correlations between phagocytosis of *Chlamydia trachomatis* (CT) EBs and IgG1 antibodies against specific CT antigens and EBs in serum samples from 60 women with CT infection outcomes measured by IgG1 ELISA at follow-up. **(A)** The percentage of cells with phagocytosed CT EBs versus the OD values of IgG1 antibodies specific to CT antigens **(B)** Correlation analysis between phagocytosis and IgG1 responses against 9 CT antigens and CT EBs (Pearson’s correlation test).

To investigate potential correlations between IgG1 responses to specific CT antigens and the functional antibody response (phagocytosis of CT EBs) that we measured using the serum samples collected from 60 women with CT infection outcomes as discussed above, we analyzed these data for these correlations. Since our correlation analyses showed no meaningful differences when analyzing data from V1 or V2 and the data from both visits cannot be combined for the analyses, we chose to present V2 data here as it is the more relevant timepoint from an adaptive response perspective. **Figure 5** illustrates significant correlations were observed with IgG1 antibodies to CT EBs (p < 0.001), MOMP (p < 0.001), Pgp3 (p < 0.001), HSP60 (p < 0.001), and PmpF (p = 0.0280). Furthermore, there was a trend towards correlations between the phagocytosis of CT EB and IgG1 antibodies specific to PmpG (p = 0.0659) and PmpH (p = 0.1040). However, no significant correlations were found between the phagocytosis of CT EBs and IgG1 antibodies targeting PmpE, AtpE, and RecO. These findings support that IgG1 antibodies that target MOMP, Pgp3, HSP60, PmpF, and possibly other potential outer membrane proteins play a role in facilitating antibody-mediated phagocytosis of CT EBs in women with CT infection outcomes.

## Discussion

Most existing CT serologic assays are limited with only low to moderate sensitivity and an inability to distinguish different CT infection outcomes. However, these assays are currently the only tests available to detect prior CT infection. In this study, we evaluated the potential of new CT serological assays to identify sensitive markers for detection of prior CT infection and to identify immune correlates of protection against CT in humans.

Our initial studies aimed to validate that the highly sensitive IgG1 ELISA methodology is a more useful tool than conventional total IgG ELISA to measure antibody responses to CT antigens. Geisler et al. previously reported that IgG1 was the predominant sustained serum immunoglobulin response to CT EBs in CT-infected humans and then further described attributes and performance of an EB ELISA that measured IgG1 responses (Geisler et al., 2012). The lower sensitivity of total CT IgG ELISA compared to IgG1 ELISA is primarily attributed to the high background reactivity observed in the total CT IgG ELISA. Consequently, a substantial dilution of serum (ranging from 1:250 to 1:1000) in the total IgG ELISA is required to reduce background responses to an acceptable value, which greatly diminishes the sensitivity of the analyses. Conversely, IgG1 ELISA utilizing monoclonal IgG1 subclass-specific detection reagents results in minimal background responses.

In ELISA, both Peroxidase (HRP) and Alkaline Phosphatase (AP) conjugates are extensively used as secondary detection reagents. In this study AP and HRP detection were used for IgG1 and total IgG ELISA assays, respectively in accordance with established protocols that have been consistently followed over many years. It is possible that HRP and AP detection methods may slightly differ in sensitivity. However, the heightened serum dilution required to reduce assay background in total IgG ELISA, as opposed to IgG1 ELISA, significantly compromises the sensitivity of the analyses.

Our results confirmed that IgG1 ELISA, even when using 1:32 diluted serum samples, yielded substantially lower background responses for CT EBs as well as 9 CT antigens of interest that we tested (the five outer membrane proteins MOMP, PmpE, PmpG, PmpF, and PmpH, well as Pgp3, HSP60 and the negative control antigens RecO and AtpE), compared to total IgG ELISA with 1:250 diluted serum samples (**Figure 1B**). Given that IgG1 is the most abundant human IgG antibody subclass in human serum, we propose that IgG1 ELISA should replace total IgG ELISA for the detection of human antibody responses to CT antigens.

Our IgG1 ELISA studies were conducted using sera from a well-characterized cohort of women with different CT infection outcomes. These outcomes included persisting infection versus spontaneous resolution documented at the time of returning for treatment of a positive CT screening test and among those with persisting infection at the time of treatment, the outcome reinfection vs. no reinfection at a 3-month follow-up visit. While IgG antibodies to some of these 9 CT antigens have been previously studied (Tan et al., 2009; Anyalechi et al., 2022; Marques et al., 2022), to our knowledge, this is the first report evaluating IgG1 responses to these antigens and comparing antibody responses to these 9 CT antigens between distinct groups with different CT infection outcomes. We found that Pgp3 IgG1 ELISA exhibited superior sensitivity for detecting IgG1 in these women with different CT infection outcomes compared to IgG1 ELISAs using other CT antigens and CT EBs. Over the past 15 years, CT Pgp3 has emerged as the most consistently targeted CT antigen in CT total IgG ELISAs (Wills et al., 2009; Horner et al., 2016; Anyalechi et al., 2022). We confirmed this finding of exceptional immunogenicity in this study by showing 97.5% IgG1 seropositivity in women with CT infection outcomes. Pgp3 is transcribed from the highly conserved CT plasmid that is not found in human *C. pneumoniae* isolates. Pgp3 exhibits a high degree of immunogenicity in its natural trimeric conformation, and antibodies targeting Pgp3 do not cross-react with *C. pneumoniae* (Horner et al., 2016). While it is possible that the 3 serum samples that tested negative for Pgp3 IgG1 simply reflect poor immunogenicity for this antigen in these CT-infected women, it is also possible these women may have been infected with a plasmid-free CT strain. A study investigating the prevalence of plasmid-bearing and plasmid-free CT infections among women found that 93.5% (86 out of 92) were infected by plasmid-bearing CT strains, while the remaining 6.5% (6 out of 92) were infected by plasmid-free strains (Yeow et al., 2016). The plasmid has been associated with virulence in animal models (Lei et al., 2014; Zhong, 2017), but its clinical significance in human CT infection is unknown. Studies on Pgp3 antibody detection in subjects with plasmid-free CT infections have not been done to our knowledge and this is worthy of further investigation considering Pgp3 ELISA is a commonly used assay for detection CT antibodies in humans.

When evaluating IgG1 responses to the 9 CT antigens and EBs in relation to CT infection outcomes, Pgp3 IgG1 responses were the only one amongst IgG1 responses to other CT antigens and EBs that showed a significant difference in magnitude of the IgG1 responses between CT infection outcome groups. A higher magnitude of Pgp3 IgG1 response was detected at V1 in the group with persisting CT infection compared to the CT resolution group and at V2 in the persisting infection group that had reinfection detected at V2, suggesting an elevated magnitude of the Pgp3 IgG1 response correlates with current CT infection. Pgp3 IgG1 antibodies are not associated with clinical correlates of protection (spontaneous resolution and absence of reinfection) and thus may not be useful as an immune correlate of protection.

Studies using the *C. muridarum* murine genital infection model have revealed that protection against reinfection is multifactorial, with CD4 T cells and antibodies playing prominent roles (Morrison et al., 2000; Morrison and Morrison, 2005). However, it has been observed that *Chlamydia*-specific antibody is unable to effectively directly neutralize *C. muridarum in vivo*. This suggests that antibody interaction with an effector cell population(s) is required for bacterial killing. Antibodies interact with immune effector populations through various mechanisms to facilitate protection against pathogens. These mechanisms include antibody-dependent cellular cytotoxicity (ADCC), typically executed by natural killer (NK) cells (Zighelboim et al., 1973), and antibody-mediated phagocytosis followed by pathogen killing by phagocytes (neutrophils and macrophages) (Schiff et al., 1997). The Morrison research group utilized the *Chlamydia* genital infection mouse model to demonstrate a protective role for both *Chlamydia*-specific IgG and neutrophils (Naglak et al., 2017), highlighting the primary contribution of neutrophils in antibody-mediated immunity. Notably, the study revealed that NK cells were dispensable for *Chlamydia* protective immunity. This compelled us to investigate antibody-mediated phagocytosis by neutrophils as a potential immune correlate of protection against CT in humans.

The flow cytometry-based assay we utilized for investigating antibody-mediated phagocytosis by neutrophils is a simple, rapid, and reproducible technique for assessing antibody-mediated phagocytosis. This method is an efficient alternative to microscopy for counting and titrating bacteria, as the microscopy approach is time-consuming, labor-intensive, and limited in its throughput. To our knowledge, we are the first group to investigate CT phagocytosis in humans with CT outcomes. We found that 95% of sera from women with CT infection outcomes exhibited positive antibody-mediated phagocytosis of CT EBs. Furthermore, we observed significant correlations between antibody-mediated phagocytosis of CT EBs and IgG1 antibodies targeting MOMP, Pgp3, HSP60, PmpF, and other potential outer membrane proteins in these women, findings which initially suggested that antibodies play a substantial role in facilitating antibody-mediated CT clearance in women infected with CT. However, as we observed for IgG1 antibodies, we did not find significant differences in antibody-mediated phagocytosis activity between the different CT infection outcomes. It is worth noting that data obtained from these *in vitro* assays may underestimate the protective role played by antibodies *in vivo*. For instance, IFN-γ-stimulated primary peritoneal neutrophils (PPNs) and non-stimulated PPNs with immune serum were incapable of reducing the bacterial burden in a phagocytic killing assay (Naglak et al., 2017). However, IFN-γ-stimulated PPNs with immune serum significantly inhibited *Chlamydia* growth, exhibiting a nearly 100-fold reduction. These findings suggest that *in vivo*, antibody-mediated protection may be achieved through IFN-γ CD4 T cell responses. Considering the influences of sera and IFN-γ on neutrophils in the murine system, the role of opsonophagocytic antibodies and neutrophils in human *Chlamydia* immunity requires further investigation.

Because the murine findings of antibody mediated protection to chlamydia reinfection are robust, we believe it is important to study mechanism for antibody mediated immunity beyond what we have studied. In addition to neutrophils, B cells (mainly B1 cells) have been previously reported to act as professional phagocytes by phagocytosing and killing bacteria (e.g., *Staphylococcus aureus, Escherichia coli*) as well as present bacterial antigens to T cells (Gao et al., 2012). B1 cells can internalize particulate antigens and extract peptides for presentation to T cells, a process that is 10^5^-fold more efficient than the presentation of soluble antigens. The phagocytic function of B1 cells may serve as an initial defense against infection and bridge innate and adaptive immunity. Therefore, investigating the phagocytosis of CT by B1 cells is important, given the potential implications for advancing the development of novel vaccination strategies against CT infection, and is a future area of research interest.

IgG exists in both systemic and mucosal circulation, contributing to long-lived production, and represents the dominant antibody isotype in reproductive tract mucosal secretions. Among IgG subclasses, IgG1 is the most abundant in human sera. We observed significant correlations among IgG1 antibody titers and phagocytosis of EBs for MOMP, Pgp3, Ct EBs, HSP60 and PmpF antigens. However, it is important to acknowledge a potential limitation in our study was that other CT-specific IgG subclasses, as well as IgM and IgA, were not specifically depleted in serum samples. Therefore, the observed high antibody:phagocytosis correlations cannot be strictly attributed to IgG1 antibodies alone. Another limitation of our study is that, while focusing on serum antibody and associated phagocytosis function, our study does not address mucosal antibody (e.g., secretory polymeric IgA) function and this is an area of future interest that warrants further exploration. Additionally, antibodies can contribute to protection through other defense mechanisms against infection, such as neutralization; the role of neutralizing antibodies in human immunity to CT deserves further investigation.

## Conclusions

IgG1 ELISA is a highly sensitive method to assess antibody responses to CT EBs and antigens. Notably, Pgp3 IgG1 ELISA exhibited superior sensitivity when compared to MOMP IgG1 and CT EB IgG1 ELISAs, justifying it as a preferred assay for CT antibody detection. Magnitude of the Pgp3 IgG1 response was highest during current CT infection, while magnitude of IgG1 response to MOMP and other outer membrane proteins we investigated did not significantly differ between our CT infection outcomes of interest. Antibody-mediated phagocytosis of CT EBs, a functional antibody response, was detected in the vast majority of women with CT infection outcomes, and opsonophagocytic antibodies correlated with the presence of IgG1 antibodies against Pgp3, HSP60, MOMP, PmpF, and other potential outer membrane proteins. While presence of IgG1 antibodies and antibody-mediated phagocytosis were detected in most women with CT infection outcomes, they were not associated with clinical correlates of protection, suggesting they may not be useful as immune correlates of protection against CT in humans.

## Data availability statement

The original contributions presented in the study are included in the article/supplementary material. Further inquiries can be directed to the corresponding author.

## Ethics statement

The studies involving humans were approved by The Institutional Review Boards of the University of Alabama at Birmingham and the Jefferson County Department of Health (JCDH). The studies were conducted in accordance with the local legislation and institutional requirements. The participants provided their written informed consent to participate in this study.

## Author contributions

HY: Formal analysis, Investigation, Methodology, Writing – original draft, Writing – review & editing. WG: Conceptualization, Funding acquisition, Investigation, Project administration, Writing – original draft, Writing – review & editing. CD: Formal analysis, Investigation, Methodology, Writing – review & editing. KG: Methodology, Project administration, Writing – review & editing. GC: Formal analysis, Writing – review & editing. RB: Conceptualization, Funding acquisition, Investigation, Supervision, Writing – original draft, Writing – review & editing.
